# Analysis on the equity differential on household healthcare financing in developing countries: empirical evidence from Tanzania, East Africa

**DOI:** 10.1186/s13561-022-00404-9

**Published:** 2022-11-07

**Authors:** Felician Andrew Kitole, Robert Michael Lihawa, Eliaza Mkuna

**Affiliations:** Department of Economics, Mzumbe University, P.O Box 5, Mzumbe, Tanzania

**Keywords:** Health Financing, Health Equity, Instrumental Variable, Health Economics, Tanzania, Developing Countries

## Abstract

**Background:**

Achieving equity in healthcare services has been a global priority. According to the literature, a slew of initiatives aimed at increasing household equity in healthcare financing have exacerbated the problem, making it hard for most developing countries to understand the real cause of the problem.

**Method:**

The non-experimental research design has been used to explore the Tanzania Panel Survey (NPS) data 2019/2020, to investigate equity differential in household healthcare financing in Tanzania by the use of conventional instrumental variable methods of Two-stage and Three-stage least square methods

**Results:**

Despite the global agenda of universal health coverage, this paper reveals that 86 percent of Tanzania lacks health insurance with a high degree of inequitable distribution of health facilities as 71.54 percent of the population is in rural areas, yet these areas have poor health systems compared to urban ones. These disparities increase pressure on household healthcare financing and widen the inequity and equality gaps simultaneously. Additionally, a household's income, education, health care waivers, out-of-pocket expenditure, and user fees have been found to have a significant impact on household equity in healthcare financing.

**Conclusion:**

To reverse the situation and increase equity in household healthcare financing in most developing countries, this paper suggests that an adequate pooling system should be used to allow more people to be covered by medical prepayment programs, and the donor-funded programs in developing countries should focus on health sector infrastructure development and not the capacity building.

## Introduction

Several studies have been conducted to analyze equity and equality in the accessibility to healthcare globally and locally [1–6]. However, all these studies face major challenges on how to apply the same concept of equity and their findings to other countries, considering that development is heterogeneous. Therefore, studies on equity need to be made on a country basis to know the extent of the problem in each country [7]. Despite equity financing being one of the global agenda towards the realization of the Sustainable development Goals (2030), yet countries differ in the way of reducing household burden due to increased disproportional financing as the poor households become more victims, which increases the demand for seeking better ways of reversing the situation [7].

Growing demands of information on health equity from the household to the national level have made local and global multilateral institutions increase research financial resources to analyze the dynamics, causes, and effects of inequity in healthcare financing from the household to the national level [1]. Additionally, equity in health financing is recently considered a significant component towards achieving universal health coverage [8].

Nonetheless, the average per person health expenditure in the world’s high-income countries is $ 4,660 while it stands at $356 in all middle-income countries and only $61 for the least developed nations [2]. Some of the SSA regions face the misallocations of resources in the health sector based on dimensions of geographical, race, social-economic difference and needs. Less attention has been paid towards equity in health care finance and expenditure which has experienced countries spending less to the Abuja declaration of 15% of GDP in the total country’s healthcare budget [6].

However, studies have shown that risk pooling, which includes incentives for the underprivileged, mandatory payments, reduces the risk of economic hardship and so helps to attain the long-term goal of universal coverage. In recent decades, most of the sub-Saharan African health have been showing promising improvement whichever been attributed to the increasing commitment of the donors in the health sector and the rise in regional’s gross domestic product [4].

After independence in 1961, the United Republic of Tanzania scrapped user fees in government health institutions. The government has embraced a policy of providing free health care [3]. This was then followed by the Arusha Declaration of 1967, which implemented several reforms in the health sector intended to ensure the poor and those living in rural areas who are marginalized have universal access to social services. To ensure equity in the provision of health care among poor people in Tanzania, the government banned private for-profit medical practice in 1977. Furthermore, through public taxes, the government took on the task of financing and delivering health care to all who people who use public health institutions [9].

Tanzania National Health Accounts (2015) and Ministry of Health reports show that the health sector finances are sourced from taxes, donors, private/NGOs, health insurance schemes, National Health Insurance Fund (NHIF), Community Health Fund (CHF), User fees and Out of Pocket Expenditure (OOP). Additionally, the composition of Tanzania’s health sector budget financing for several years has left households vulnerable by being a large financier of the total sector budget. In financial year (FY) 2002/2003 the composition was characterized by public sector 25%, development partners 27%, households 42% and other private 5% in FY 2002/03 [8]. In FY 2005/06 public sector contributed 28%, development partners 44%, households 25% and other private 3% [8]. In FY 2009/10 public sector contributed 26%, development partners 40%, households 32% and other private 2%. Moreover in 2011/12 public sector contributed 22%, development partners 48%, households 25% and other private 4% in FY 2011/12 [10]. In cementing these facts, the literature argues that nearly half of health system financing in developing countries comes from households [3].

Tanzania's government committed TZS 2,222 billion towards the health sector in FY 2017/18, accounting for 7.0 percent of the entire budget [11, 12]. Apart from the country’s economic growth in recent times, development partners contribute an average of 4 US dollars for every 10 US dollars spent in the Tanzania health sector. To emphasize the donors’ contributions to the health sector in Tanzania, reports shown that in FY 2010/11, the share of government expenditure on the health sector was just 11.9 percent below the 15 percent thresholds of the Abuja declaration [5]. Apart from donors' contributions, households in Tanzania are the largest funders of health services through different payment schemes initiated by the government, including - user fees [8, 11].

To reduce the household level burden due to these health service schemes, the government initiated several programs and policy options intending to increase accessibility to health care services, reducing inequities in health and widening citizens' ability to access healthcare services in their residential areas [12].

Despite Tanzania’s government investment in different health strategies, - equity in healthcare financing has been a nightmare and many studies have shown that these strategies have been deepening the problem instead of lessening it [13–17]. Hence, a large burden has been placed on poor households in rural and urban areas due to insufficient government healthcare financing. Moreover, these studies have been based on normative measurement of equity and failed to capture the most important components that affect equity in healthcare financing, which provides biased estimations. Therefore, this paper examines the nature and disparities of equity in healthcare financing among households in Tanzania. Understanding such a link will help to design policies that take into account the difference in household socioeconomic characteristics, particularly income, which has been a major determinant for the household decision to finance health care services. This creates divergence and inequities among rich and poor groups in Tanzania and other similar countries.

## Theoretical Foundation and Modeling

Estimating equity in health care financing involves analyzing both horizontal and vertical equity in health care [18]. The model quantifies and tests for violations of the principle of ‘equal treatment for equal need’ that are related to socioeconomic status resulting from individuals’ income. This study improves this model by modelling the household financing levels of equity based on international recommendations to be able to capture all households based on their levels of equity and not just consider households to be homogeneous.

In this study, the equity was determined by the use of the Theil index (TI) proposed by the Dutch economist H. Theil who used entropy to determine unfairness of income. The TI ranges between 0 and 1. Although the TI was originally proposed as a measure of income inequality it is now common measures of disparity in health research, and of inequity in health resource allocation [19].

1ai $$T=\sum \limits_{i=1}^n{P}_i\log \frac{P_i}{Y_i}$$

Thus, *P*_*i*_ is the percentage of area’s population from the total population while *Y*_*i*_ is the proportion of health resources owned by the areas’ accounts from the total number of health resources; this leads to the expansion of the decomposition formula;

1aii $$T={T}_{\mathit{\operatorname{int}} ra- class}+\kern0.5em {T}_{\operatorname{int} er- class}$$

Whereas,

1aiii $${\displaystyle \begin{array}{cc}{T}_{\mathit{\operatorname{int}} ra- class}=& \sum \limits_{g=1}^k{P}_g{P}_g\end{array}}$$

1aiv $${\displaystyle \begin{array}{cc}{T}_{\mathit{\operatorname{int}} er- class}& =\sum \limits_{g=1}^k{P}_g\log \frac{P_g}{Y_g}\end{array}}$$

Therefore, as used in this study, the above formulas explain that *T*_*intra* ‐ *class*_ is the difference of health allocated resources within the region while *T*_*inter* ‐ *class*_ means differences of health resources between regions in Tanzania, therefore to capture the inequity at the household level the computation have based on the expenditure patterns of households in the healthcare services.

### Household healthcare financing model

The unitary household model for consumer behaviour presumes that when households behave like it maximizes a sole price independently social benefits function is subjected to family budget constraints [20–22]. Households’ choices are jointly determined which implies that individual members of a household have similar preferences [23].

Therefore, by utilizing the utility function, consider a household with members $${\displaystyle \begin{array}{cc}i\kern0.5em \in & \left[1,2\dots \dots ..n\right]\end{array}}$$ facing the utility-maximizing problem at Eq. 1b:

Subject to

1b $${\displaystyle \begin{array}{ccc}Y=& {\sum}_{i=1}^n{l}_i{w}_i+& {\sum}_{i=1}^n{y}_i{y}_j\end{array}}$$

Equation (1) explains that, household members whose characteristics are given by *h* are assumed to derive utility from the consumption of good *c.* Moreover*,* total household income *Y* is the sum of incomes earned by each member of the household ( *l*_*i*_*w*_*i*_and *y*_*i*_) and income [8] earned by the household members jointly *y*_*j*_.

Additionally, *l*_*i*_*w*_*i*_ represents labour income consisting of salaries and wages and *y*_*j*_ the non-labour income such as dividends, interest, inheritances, government assistance or transfer of payment^1^ and other waiver benefits offered by the government. Assuming income is pooled and individual members in a household have identical preferences, the solution to this utility maximization problem yields the household consumption function in terms of price *p,* total household income, *Y* and the household characteristics, *h* as shown in Eq. 2.

2 $${\displaystyle \begin{array}{cc}{c}_1=& f\left(p,Y,h\right)\end{array}}$$

Therefore, one of the limitations of unitary models is on the collective households model which has been developed with the assumption that individual member of households has a utility of

$${\displaystyle \begin{array}{cc}{\tilde{U}}^i=& {\tilde{u}}^i\left({c}^1,c2\dots \kern0.5em \dots {c}^n,h\right)\end{array}}$$ where $${\displaystyle \begin{array}{cc}{c}^s=& \left(s=1,2,..\dots n\right)\end{array}}$$ and *h* symbolize the consumptions functions of an individual together with characteristics of the household simultaneously [18].

On the other hand, the Pareto optimality for the household member with *s* is obtained by calculating below given maximization utility function;

3 $${\displaystyle \begin{array}{l}\begin{array}{cc}& \end{array}\\ {}\begin{array}{ccc} MaxW=& W\left[{\tilde{u}}^1\left({c}^1,{c}^2\dots \kern0.5em \dots {c}^n,h\right)\right.\dots \kern0.5em .\dots & {\tilde{u}}^n\left({c}^1,{c}^2\dots \kern0.5em \dots {c}^n,h\right)\end{array}\end{array}}$$

Or can be summarized as;

4 $${\displaystyle \begin{array}{cc} MaxW=& W\left[{\tilde{u}}^1..\dots, {\tilde{u}}^n\right]\end{array}}$$

*W*[⋅] is a household aggregate prices dependent utility function with its maximization is normally subjected to household budget constraints. The budgetary constraints are a monotonicity function of utility for every member of a household, ${\tilde{u}}^i$ to ${\tilde{u}}^n$ and the solution to these maximization problems are always functions of Marshallian demand to every member of the household and covariates these demand functions are individual and shared incomes, price as well as the characteristics of the household.

However, the general function will be represented by the hereunder function with some of the important variables

5 $${\displaystyle \begin{array}{cc}{HHCF}_i=& f\left({Userfee}_i, Dfactors,{OOP}_i,{Exemption}_i, Sfactors\right)\end{array}}$$

where *HHCF*_*i*_ is the healthcare financing in *i*^*th*^ household *Userfee*_*i*_ is the fee payment made by the household during healthcare utilization, *OOP*_*i*_ is the out of pocket expenditure incurred by households, and *Exemption*_*i*_ include all free services whose costs are covered by the government to the vulnerable groups in society including elders, maternal and under-five children, *Dfactors* are demographic factors and *Sfactors* are socioeconomic factors including education and income.

## Methodology and data

### Data type and source

This study uses a statistical study design to analyze secondary data of the Tanzania National Panel Survey (NPS) 2019 – 2020 sourced from the World Bank [24]. World Bank is a credible data centre; therefore this study introduces estimations from such sources, which increases reliability and accuracy of the information provided in this paper. Moreover, the NPS contains information on the household socioeconomic statuses including household health, income and expenditure patterns. Specifically, health information collected are those concerned with general health status and utilization of health services; source and financing of health treatments/hospitalization, disaggregated health expenditures, disability, bednet use, pregnancy, prenatal care and births, child health and ailments/diarrhea.

### Data analysis

This paper has employed the use of the Two-Stage Least Square method (2SLS) and Three Stage Least Square models (3SLS); the decision to use these models has been based on the possible endogenous between health equity and household health care financing when estimating the effects of health equity on household health care financing at Eq. 6.

6 $${\displaystyle \begin{array}{ccc}\ln \kern0.5em {HHCF}_i=& {\beta}_1{HE}_i+& \sum \limits_{j=2}^n{\beta}_j\kern0.5em {h}_i+{\varepsilon}_1\end{array}}$$

7 $${\displaystyle \begin{array}{cc} HE=& \sum \limits_{i=1}^n{\alpha}_1\kern0.5em {h}_j+{\varepsilon}_2\end{array}}$$

Where ln *HHCF*_*i*_ is a log of household health financing, *HE*_*i*_ is a health equity variable that captures household health equity rank, and *h*_*i*_ is a vector of exogenous variables while *h*_*j*_ is a vector of exogenous variables consisting of instrumental variables that affect health equity but have no significant effect on household income. On the other hand, *h*_*i*_ are covariates belonging to the income Eq. 6, *β*_1_, *β*_*j*_ and *α*_1_ are parameters to be estimated while *ε*_1_ and *ε*_2_ are the disturbance terms. Two-stage least squares method was applied in estimating Eq. 6 whose results have been presented in Column 2 of Table 5 of which the instrument used to identify Eq. 4 was the distance to the nearest health facility. Moreover, a series of tests have been made to test assumptions of the model, its strengths as well as the validity of the used instrument of which by substituting Eq. 7 into Eq. 6 we obtain the new Eq. 8:

8 $${\displaystyle \begin{array}{ccc}\ln \kern0.5em {HHCF}_i=& {\beta}_1\sum \limits_{i=1}^n{\alpha}_1\kern0.5em {h}_j+& \sum \limits_{j=2}^n{\beta}_j\kern0.5em {h}_i+{\mu}_i\end{array}}$$

Thus, Eq. 6 is referred to as the structural form while Eq. 7 and 8 are as first stage and reduced form respectively while the error term *μ*_*i*_ is an invertible linear transformation of *ε*_1_ and *ε*_2_.

## Discussion of findings

### Descriptive statistics

The descriptive statistics of the key research variables show variations in the number of households’ characteristics as analyzed in Tables 1 and 2. Therefore, descriptive analysis has been used to describe the characteristics or nature of the respondents in the study. It summarizes the collected and analyzed data. This analysis provided a quantitative virtual understanding of the data using statistical methods.

**Table 1:** Head of household characteristics

Characteristics	Categories	Frequency	Percentage
Residence	Rural	6,770	71.54%
	Urban	2,693	28.46%
Sex	Male	7,111	75.15%
	Female	2,352	24.85%
Level of Education	No Formal Education	1,905	20.13%
	Primary Education	6,153	65.02%
	Secondary Education	702	7.42%
	Higher Education	703	7.43%
Marital Status	Married	5,160	54.53%
	Not Married (Otherwise)	4,303	45.47%

**Table 2:** Household Characteristics on Key Variables

Characteristics	Categories	Frequency	Percentage
Employment Status	Employed	3,875	40.95%
	Otherwise	5,588	59.05%
Improved Sanitation	Yes	6,585	69.59%
	No	2,878	30.41%
Healthcare Waiver	Waiver (Exempted)	4,897	51.74%
	Otherwise	4,566	48.26%

Results in Table 1 show that 6,770 respondents equivalent to 71.54% of the entire respondents are residing in rural areas while only 2,693 equivalent to 28.46% are residing in urban areas. Moreover, out of 9,463 households; maleheaded households were 7,111 (75.15%) while female-headed households were 2,352 (24.85%). In addition to that, respondents’ level of education differs significantly across all households whereas 20.13% had no formal education, 65.02% attended primary education and those who attended secondary and higher education were 7.42% and 7.43% respectively. Findings further reveal that married couples were 5,356 equivalents to 54.53% while those who had not been married were 45.47%.

Findings in Table 2 indicate that 3,875 head of households were employed in different economic activities many being farmers and other petty economic activities that were equal to 40.95% while 59.05% were found not employed in any economic activities. In analyzing the household level of health, the study found that out of 9,463 households only 30.41% were not having access to improved sanitation. Nonetheless, 51.74% of all households were found to have healthcare waiver cards provided by local authorities. These waivers are for special and vulnerable groups while only 48.26% were found to have no healthcare waiver or benefits. These waivers are due to the Tanzania health policy and are funded by the central government.

**Fig. 1:**
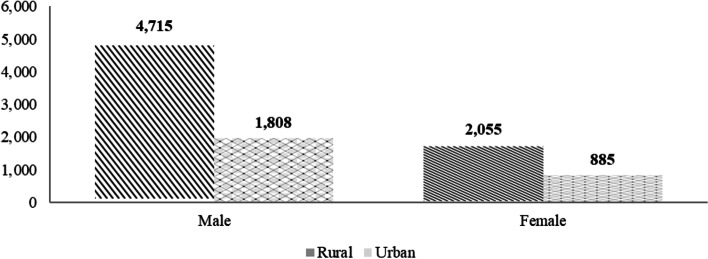
Residence occupancy based on gender

Findings in Fig. 1 demonstrate that out of 6,770 households that are living in rural areas; the gender distribution was 4,715 males and 2,055 females, equivalent to 69.64% and 30.36%, respectively, while in an urban areas, the distribution was 67.15% for males and 32.85% for females Fig. 3.

Findings in Table 3 demonstrate that the average age of all respondents was 48 years while the average household size for 9,463 households was 6 people. The average household healthcare expenditure across all households was Tshs 455,000.1 with an average household’s income of Tshs 957,356. The average amount of money used by each household as a user fee for the utilization of the healthcare services across all households was Tshs 150,260.2 while the average out-of-pocket (OOP) expenditure across all households was Tshs 8,685.33.

**Table 3:** Socioeconomic Characteristics of Households

Variables	Minimum	Maximum	Mean	Standard deviation
Age	21	114	48.03585	18.24101
Household Size	1	36	6.033711	4.21865
Total Health Expenditure	4,325	1,500,000	455,000.1	1,690,302
User fee	0	4,614,256	150,260.2	148,132
Household Income	19,430	1,210,420	957,356	1,727,094
Out of Pocket Expenditure	0	2,160,000	8,685.33	42,777.19

**Fig. 2:**
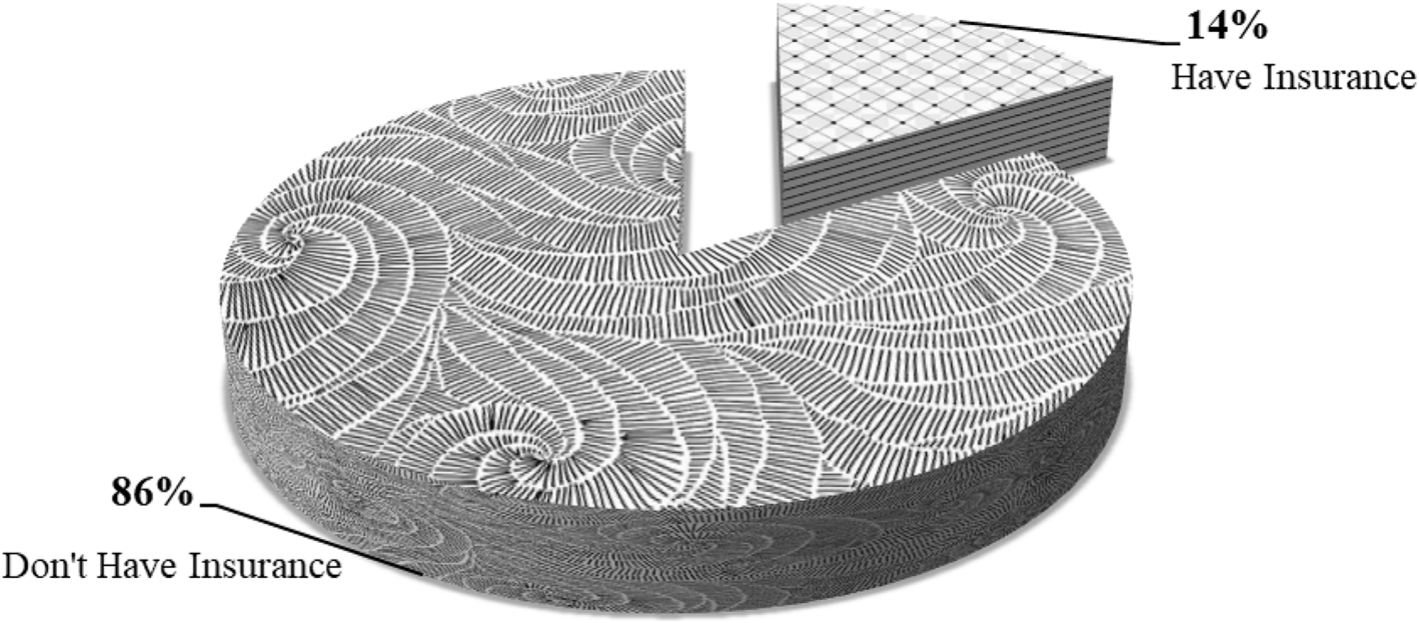
Distribution of household insurance cover

The results in Fig. 2 show that 86% of the entire households do not have health insurance while only 14% are insured out of 9,463 households. This implies that the majority of households have to pay through user fees and largely the dominance of the out-of-pocket expenditure (OOP).

**Fig. 3:**
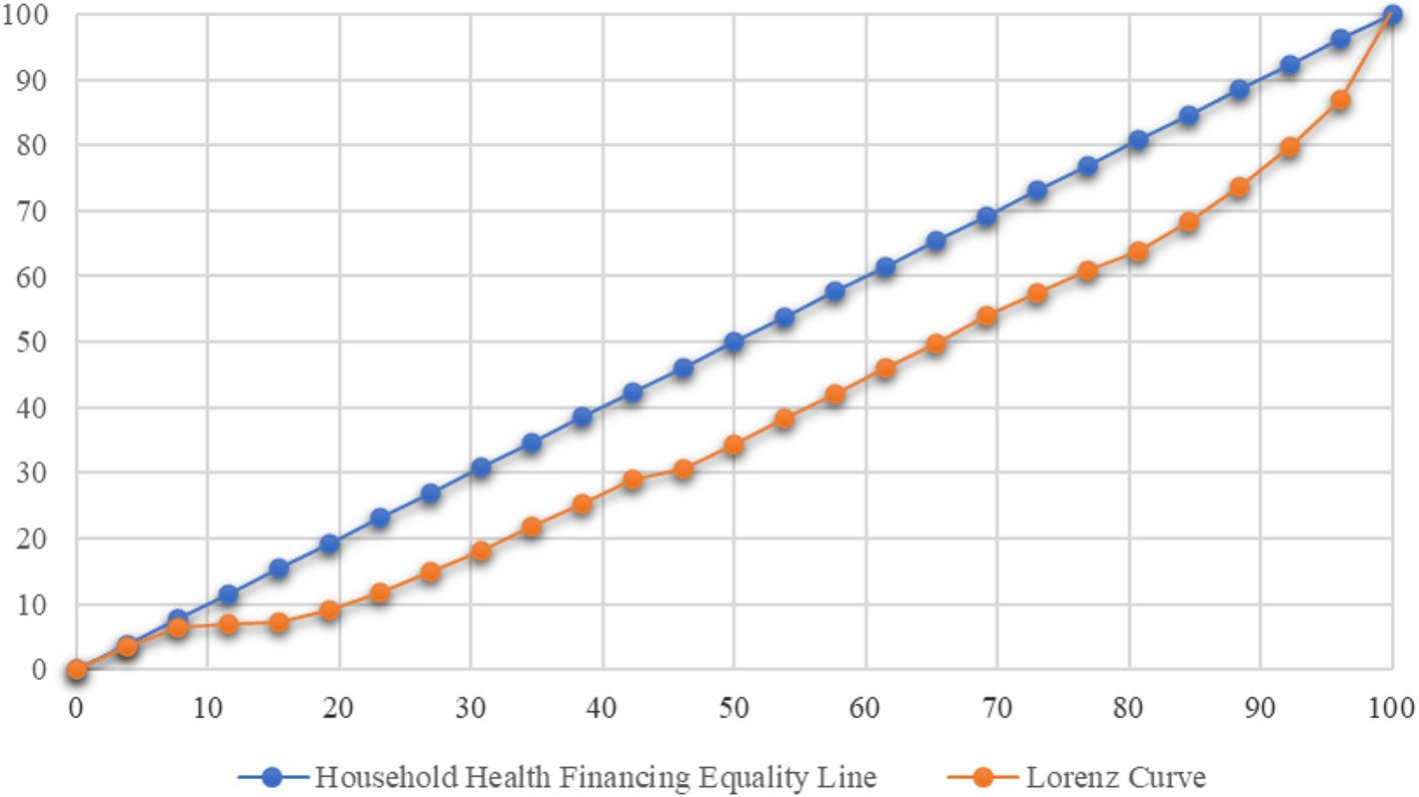
Lorenz health resource curve for household healthcare financing

Findings in Table 4 show that a total of 8,138 households do not have health insurance which is approximately 86% of all households. However, out of these households with no health insurance coverage 72.25% are living under the lowest equity level (inequity) with a low ability to finance healthcare services. These results justify that having health insurance improves household equity in covering and financing health services, as indicated by the fact that only 40 households out of 1,325 with health insurance are living under the lowest equity of financing health services, which is just 3.02% of the whole households with health insurance and just 0.42% of the whole 9,463 households (Table 4).

**Table 4:** Equity among insurance-covered households in Tanzania

Health Equity	Insurance Cover		Total
	Don’t Have Insurance	Have Insurance	
Lowest Equity (Inequity)	5,880	40	5,920
Low Equity	1,118	124	1,242
Moderate Equity	953	733	1,686
Highest Equity	187	428	615
**Total**	**8,138**	**1,325**	**9,463**

The regressionoutputs in Table 5 present the estimated coefficient of parameters used in Eqs. 6 and 7 by analyzing the effects of equity on household healthcare financingwhereas the distance to the nearest health facility was used as an instrument. Therefore, findings revealed that having formal education increases the likelihood of increasing household healthcare financing by household to spend more money on health care services compared to heads of households with no formal education. The relationship between formal education and health expenditure is positive at a 1% level of significance. Moreover, waiver programs implemented by the government in health care services have been found to influence an increase in the household healthcare financing positively and significantly; thus as households having healthcare exemption increases its healthcare financing by 57.118% compared to those that have no exemption cards for medical treatments.

**Table 5:** Effects of equity in household healthcare financing in Tanzania (2SLS)

Variables	OLS	Instrumental Variables Regression (2SLS)
Health Equity	-0.1243365** (0.0214821)	-0.1973647*** (0.0043935)
Education level (Formal education)	0.1229195***	0.39924***
	(0.01395686)	(0.028312)
Location (Urban)	0.1278835***	0.377***
	(0.01706361)	(0.015402)
Sex (Male)	0.1781712**	-0.0997***
	(0.07784514)	(0.0158)
Age	-0.08926049*	0.0114***
	(0.001191912)	(0.0024101)
Age square	0.07600633 *	-0.000108***
	(0.00119985)	(0.0000227)
User fee	-0.1119285**	-0.31806***
	(0.0030004)	(0.061508)
Insurance	-0.4686392**	-0.266***
	(0.09466508)	(0.19212)
Out of pocket	-0.1024091***	-0.235701***
	(0.0072669)	(0.100147)
Household size	0.09530194*	0.10537***
	(0.0063909)	(0.00215)
Exemption (waiver)	-0.5711851***	-0.27765***
	(0.1419761)	(0.019230)
Household income	0.1094737**	0.19541***
	(0.01908869)	(0.003212)
Observations	9,398	9,398
R-squared	0.7695	0.6269
Instruments	Distance to the nearest health facility	
Instrumented	Household Healthcare financing	

Standard errors in parentheses

*** *p*<0.01, ** *p*<0.05, * *p*<0.1

Additionally, the study has found that household healthcare financing is significantly reduced by the prevalence of user fees, insurance and out-of-pocket expenditure as all three variables have a negative relationship with household healthcare financing. Specifically, findings revealed that monetary increase in the health care user fees reduce household ability to finance health care by 31.806% while out-of-pocket expenditure depletes household healthcare financing ability by about 23.5701%. On the other hand, households having health insurance were found to reduce household healthcare financing by 26.6% compared to those who have no health insurance.

On top of that, having formal education has been found to increase household healthcare financing by 39.924% significantly. However, being a beneficiary of government health care waiver programs reduces household spending in financing healthcare services by 27.765% significantly. Lastly, results in Table 5 show that an increase in the household size increases the likelihood of the household to finance and spend more of its income on healthcare financing by 10.537% significantly. Similar results have been found on the household income that, an increase in household income increases household healthcare financing by 19.541%.

The 3SLS method has been used to analyze three different equations as seen in Table 6. The results obtained after the 3SLS showed that formal education had similar effects in increasing equity in household financing significantly. Similarly, the 3SLS had shown significant effects of urban and sex of head of households towards equity in healthcare financing. Moreover, findings in Table 6 show that a monetary increase in the user fee charges diminishes equity in health care services by 36.206%. On the other hand, a monetary increase in the out-of-pocket expenditure reduces the equity in health care services by 22.480% significantly.

**Table 6:** Three stage least square method

Endogenous variables	Exogenous variables	Coefficient
Health Equity	Formal education	0.3953772*** (0.0282077)
	Location (Urban)	0.3730041*** (0.0154001)
	Sex	0.0999472*** (0.0157308)
	Age	-0.0116181 (0. 2024077)
	Age square	0.0001098 (0. 1000226)
	User fee	-0.36206*** (0.0006108)
	Insurance	0.2644691*** (0.0191129)
	Out of pocket	-0.2248006*** (0.0140007)
	Household size	0.063415** (0.0021516)
	Exemption (Waiver)	0.0871979*** (0.0189715)
Household Health care Financing	Household income	0.2010511*** (0 .0007881)
	Employment Status	0.2409207*** (0. 1109457)
	Household Food consumption	-0.1870436*** (0.034591)
	Household non-food consumption	-0.132808*** (0.0026456)
	Exemption (Waiver)	-0.147131*** (0.00411)
	Out of pocket	0.1948006*** (0.0140007)
Household Sanitation	Employment status	0.1669335* (0.4861792)
	Formal education	0.2193210*** (0.018101)
	Location (Urban)	0.271001*** (0.0224002)
	Sex	0.0999472*** (0.0157308)
**Summary of Equations**		
**Equations**	**Observations**	**R square**
Health Equity	9,398	0.3979
Household Health care Financing	9,398	0.7356
Household Sanitation	9,398	0.3202

Standard errors in parentheses

*** *p*<0.01, ** *p*<0.05, * *p*<0.1

On the other hand, findings in Table 6 further show that household income and employment status of the head of household have been found to increase healthcare financing by 20.105% and 24.092% significantly while an increase in household expenditure on food and non-food have been found to reduce household healthcare financing by 18.704% and 13.281% respectively. Additionally, households that have been exempted from paying some of the health care costs due to national policies have been found to have less costs of healthcare financing compared to those who are not exempted from any health care services. The out-of-pocket expenditure has been found to increase the household health care financing burden by 19.480%.

The TI index indicate that the lower the TI index the more there is unequal distribution, therefore the lower calculated TI herein indicate that there is higher healthcare financing gap among households in Tanzania which is justified by the TI values of 0.1042^2^ which is equivalent to 10.42% justifying that only 10.42% households are living near the line of equality of health care financing while 89.58% are far below the line of equality.

### Model Specification

The question of whether there is possible endogeneity is not a matter of researcher intuition but rather brought out of the econometrics test, which in this study has been tested during estimating (Eqs. 6 and 7). Therefore, the Hausman Test for endogeneity has been used to test the hypothesis that *H*_0_ : *Variables are exogeneous*.

Results in Table 7 justify the presence of endogeneity because the rule of thumb for a test allows researchers to reject the null hypothesis when the test p values are significant. Therefore, we reject the null hypothesis that variables are exogenous and therefore conclude there was endogeneity during estimating Eqs. 6 and 7. Hence the approach used in this study to suppress the endogeneity is correctly specified making the estimation unbiased and consistent.

**Table 7:** Tests of endogeneity

Tests	Test Scores	***P*** - Value
Durbin (score) chi 2 (1)	503.685	0.0000
Wu-Hausman F (1, 9398)	531. 473	0.0000

### Strength of instruments

The strength of the instrument has been analyzed from the two-stage least square point of view; this is normally done to identify how powerful the instrument was during the first and second regression. In this study, distance to the nearest health facility is referred to as the instrumental variable because it satisfies three important conditions that it has causal effects on the occurrence of other exogeneous variables; it affects the outcome variable only through its effects on the exogenous meaning that it has no direct influence on the outcome variable which justifies the exclusion restriction assumption; and lastly, in this study, the instrument has no confounding effects on the outcome variable [21, 25].

Moreover, when instruments are weakly correlated with the endogenous regressors, the Two stage least square (2SLS) becomes unreliable. In particular, the IV estimators can be badly biased, the t-test fails to control the size, and the conventional IV confidence interval may cover the true parameter's value less often than we intend. The most famous tests to identify weak instruments (strength of instruments) are first Stage F-statistics (Table 8) and Eigenvalue Statistic (Table 9).

**Table 8:** First-stage regression summary statistics

Variable	R-square	Adjusted R-square	Partial R-square	F(3, 9398)	Prob > F
Distance to the nearest health facility	0.7695	0.7693	0.6691	8.2e+06	0.0000

**Table 9:** Instrument Strength by Eigenvalue Statistic

	Critical Values			
2SLS relative bias	5%	10%	20%	30%
	13.91	9.08	6.46	5.39
	10%	15%	20%	25%
2SLS Size of nominal 5% Wald test	22.30	12.83	9.54	7.80
LIML Size of nominal 5% Wald test	6.46	4.36	3.69	3.32

**eigenvalue statistic = 82.32**

*H*_0_ : *Instruments are weak*

Therefore, based on the results in Table 8 shows that the whole model used in the first regression is good as it has the R square of 0.7695 (76.95%) which is significant even at 1% level. Additionally, at the first stage, the F statistic is larger than the rule of thumb of 10 hence the instrument is not weak [25]. Normally in the F-statistic, the model is considered weak if it has a lower F statistic value of which in our case the F statistic is very large signaling that the model is not weak.

A single test on the strength of the instrument was not enough to justify the strength of the instrument used, therefore we applied the eigenvalue statistics test which uses the Limited Information Maximum Likelihood (LIML) method whose results have been presented in Table 9. Thus, since the eigenvalue statistic is greater than all critical values in Table 9 even at 5% we hereby reject the null hypothesis that instruments are weak. Therefore, based on this test, we don’t have a weak instrument problem at all, confirming that the instrument used does not have any direct effect on the outcome variable but rather has only indirect effects through the treatment variable [21, 25].

### Discussion and policy implications

Equity in health care services provision is a major concern of many countries. Various social, economic, and political situations have produced diverse viewpoints on how to address the equity issue at the household and the global level. In Africa, some literature has found that user fees reduced the level of equity not only in healthcare financing but also in healthcare utilization among households in African countries such as Burkina Faso, Burundi, Ghana, Tanzania, and Kenya [26]. Unlike in West Africa where studies report that the introduction of user fees contributed to the improvement of the health services and narrowed inequity [27]. Since, user fees have different results in different countries, precautionary measures have to be taken regularly to ensure it would not affect citizens' healthcare utilization and worsen the situation.

Therefore, this controversy, discloses important information for the health systems in developing countries that inequity in healthcare finance and access to services is not just due to user costs, but the alternative finance options aimed at lowering total out-of-pocket expenses are critical to increasing equity in healthcare financing [27]. Health insurance coverage has been significantly found to influence equity improvement among households [3]. In that case, the government has a role to play in facilitating easily accessibility of affordable health insurance in rural areas, where insurance are rarely possessed and used by residents compared to urban areas.

Moreover, the ideal situation of universal insurance coverage cannot be attained within a short period, especially in a developing state like Tanzania. Even in high-income countries, the transition lasted for many years (decades) therefore this is more likely for low-income countries including Tanzania. Therefore, in the road towards universal health insurance, different schemes of payments should be used especially those focusing on the community’s socioeconomic statuses like Community Health Insurance Fund (CHIF). There is no single global path that can be used to achieve equity in healthcare financing as most of the techniques depend on the government budget and culture and historical perspective of countries as well as the institutional set-up of countries. Moreover, all these require good governance and political willingness in increasing expenditure in health care initiatives. Therefore, a continual concern should be kept on building a strong health system that will ensure equitably health benefits to all income groups in a country [21].

Nonetheless, results have shown that, households with large family size spent a lot in healthcare financing compared to those with small family size, moreover, the more the household incur high costs in health financing the more it deteriorates in terms of equity in health care. In this regard, there is a need for policymakers to introduce and increase political willingness in these programs aimed at reducing or discouraging households from having many children. In doing so the government will be able to reduce even costs of health sector financing in the long run as well as encourage equity among households towards health financing and expenditure.

## Conclusion

Development heterogeneity among countries has deepened across provinces, especially in the developing world which experiences greater development inequality between rural and urban provinces (regions). These intra-development inequalities have made rural residents more vulnerable to diseases and poor health with little attention paid by the government in reverting the situation as most governments have focused on urban that create more revenues for these countries. The results of this study, therefore, recommend that health insurance is an important component in promoting equity in health care financing among households. These justification signal government efforts to improve the national and community health insurances that are easily affordable to a large group of the society. The importance of health insurance has also been highlighted in this study as an alternate measure to lower the effects of user fees and out-of-pocket expenditures that negatively affect both household healthcare financing and health equity.

Additionally, the government policies of providing waivers to some of the special groups (elderly, children, and pregnant women) as done in most of the developing countries have been found to increase equity in health care services and reduce household's burden on health care financing. Therefore, by improving these waivers and enlarging their scope to reach a large number of underprivileged people and the poor, more developing countries like Tanzania will be able to improve health equity while lower the household economic burden as a result of increased household health care financing costs.

On the other hand, since health equity has been found to lower health care financing costs among households, the government and policy makers have to implement health care programs that promote just and equality among all groups of people. The underprivileged groups should be given extra attention so they can be able to utilize health care services in the same way the rich or high-income earners are enjoying their economies of scale in health. Equality in health may not reflect the reality of problems in the health sector, therefore investing in improving health infrastructure to be accessed by low-income earners and underprivileged groups will help to increase equity and lower health care costs burden that can prone households to impoverishment.

Finally, most of the donor-funded projects and monies should be used in the health sector’s infrastructure development and not on capacity building. Investing in infrastructure will force developing countries to expand their health services and employ more health workers in different health facilities, especially in rural areas where households have been reported spending a lot on health and even travelling long distances just to seek medical care services.

## Data Availability

Data and materials will be available upon reasonable request

## Abbreviations

CHIF: Community Health Insurance Fund
CHF: Community Health Fund
HHCF: Household Healthcare Financing
LIML: Limited Information Maximum Likelihood
NHIF: National Health Insurance Fund
OOP: Out of Pocket
TI: Theil Index
SSA: Sub-Sahara Africa
FY: Financial year

## Footnotes

1 These are payments provided by the government to special groups

2 $${\displaystyle \begin{array}{ccc}T=& {\sum}_{i=1}^n{P}_i\;\log \frac{P_i}{Y_i}=& 0.1042\end{array}}$$
